# Quality of Prenatal Care and Associated Factors among Pregnant Women at Public Health Facilities of Wogera District, Northwest Ethiopia

**DOI:** 10.1155/2020/9592124

**Published:** 2020-01-29

**Authors:** Asrat Kassaw, Ayal Debie, Demiss Mulatu Geberu

**Affiliations:** ^1^Ebinat District Health Office, Maternal Health Department, Amhara National Regional State, Ebinat, Ethiopia; ^2^Department of Health Systems and Policy, University of Gondar, College of Medicine and Health Sciences, Gondar, Ethiopia

## Abstract

**Background:**

Prenatal care refers to services a pregnant woman receives during pregnancy to ensure a healthy outcome for herself and her newborn. However, only limited studies have so far been done to assess the quality of prenatal care in the study area. Thus, this study is aimed at assessing the quality of prenatal care and associated factors at public health facilities in Wogera district, northwest Ethiopia.

**Methods:**

An institution-based cross-sectional study was conducted in Wogera district from March to April 2019. A total of 465 pregnant women were interviewed using a semi-structured interviewer-administered questionnaire; consecutive sampling was used to select the participants. The binary logistic regression analysis model was fitted to identify the potential predictor variables. Variables with <0.2 *p* values were fitted into the multivariable logistic regression analysis model; <0.05 *p* values and an adjusted odds ratio (AOR) with a 95% confidence interval (CI) were used to declare factors associated with the quality of prenatal care.

**Results:**

The overall quality of prenatal care was 32.7% (95% CI: 28.1, 37.2). Four or more prenatal care visits (AOR = 2.3; 95% CI: 1.2, 4.7), high maternal education (AOR = 2.9; 95% CI: 1.03, 7.93), over USD 175.5 monthly household income (AOR = 2.8; 95% CI: 1.1, 7.8), and the availability of maternity waiting areas (AOR = 2.4; 95% CI: 1.2, 5.0) were positively associated with the quality of the care.

**Conclusion:**

The overall quality of prenatal care in this study was low. Therefore, promoting focused prenatal care and increasing infrastructure, encouraging maternal education, and compensating for the healthcare costs for women with low household income might enhance the quality of the care.

## 1. Introduction

Pregnancy is one of the most important events that constitute a powerful experience in the lives of women and their families. Although pregnancy is a normal physiological process, it has health risks for the women and the newborns [1]. Globally, complications during pregnancy, childbirth, and the postnatal period have been the leading causes of death and disability among reproductive age women [2]. As a result, 10.7 million maternal deaths are reported to occur before the age of 25 years globally. Thus, 2.7 million neonatal deaths and 2.6 million stillbirths were reported in the world from 1990 to 2015 [3]. Additionally, nearly 99% of the maternal deaths occurred in low- and middle-income countries, particularly in South Asia and Sub-Saharan Africa [2, 4]. Although the proportion of women attending prenatal care was satisfactory, maternal and neonatal mortality remains high in the region [5].

Maternal deaths can be reduced if women can access quality medical care during pregnancy, childbirth, and postpartum [3, 6]. EDHS 2016 showed that the maternal mortality ratio (MMR) in Ethiopia was 412 per 100,000 live births [7]. This can be alleviated by quality prenatal care that reduces poor birth outcomes through increasing the utilization of institutional delivery [8, 9]. However, the proportion of women who made just one and four or more prenatal care visits did not exceed 32 and 62%, respectively, and only 26% of such women delivered at institutions [7].

Antenatal care or prenatal care is the service provided to pregnant women in order to ensure the best health conditions for the women and the fetuses during pregnancy [10]. The quality of prenatal care has an important role in the prevention, monitoring, early detection, and treatment of maternal health problems, enhancing maternal satisfaction, and healthcare utilization [11, 12].

Previous studies showed that pregnant women who received quality prenatal care were 4.6 to 47.1% globally and 24.3 to 69.5% in Ethiopia [13–15]. However, the few available studies are inadequate for generalizations because the studies used the Donabedian Model for measuring the quality of care [16]. The quality of prenatal care can be measured using a six-dimension quality of care measurement tool, namely, “information sharing, anticipatory guidance, provision of sufficient time, respect and support, availability, and approachability” [17]. Therefore, this study is aimed at assessing the quality of prenatal care using the above quality of care measurement dimensions and associated factors at public health facilities in Wogera district, northwest Ethiopia.

## 2. Materials and Methods

### 2.1. Study Design and Settings

An institution-based cross-sectional study was conducted at public health facilities of Wogera district, Amhara National Regional State, northwest Ethiopia, from March to April 2019. Wogera district is 778 km from Addis Ababa the capital of Ethiopia and 220 km from Bahir Dar the capital of the Amhara region. According to the 2007 Central Statistical Agency of Ethiopia (CSA), the district had a total population of 220,566. 112,445 of whom were males, and 8,080 women were estimated to be pregnant [18]). The district had 8 health centers, one primary hospital, 41 health posts, 4 primary private clinics, and 2 drug stores.

### 2.2. Population and Sampling Procedure

All pregnant women taking prenatal care at selected public health facilities of Wogera district were the study population. The sample size was calculated using the single population proportion formula with an assumption of 95% confidence level, 24.3% proportion of pregnant women receiving good quality prenatal care [13], 5% margin of error (*w*), 1.5 design effect, and 10% nonresponse rate which yielded a final sample of 468. Subsequently, three health centers, namely, Amba Giorgis, Gedebiye, and Tirgosige were selected using the lottery method, and Wogera hospital was selected purposively. A proportional allocation of the sample to the selected health facilities was made based on the reports of their previous month prenatal care attendants, while the consecutive sampling technique was used to select the participants.

### 2.3. Variables and Measurements

Antenatal or prenatal care visit is a healthcare visit of pregnant women in order to receive the routine control of the presumed health of pregnant women; diagnose diseases or complicating obstetric conditions without symptoms; and inform about lifestyle, pregnancy, and delivery [19].

The quality of prenatal care was measured by information sharing, anticipatory guidance, the provision of sufficient time, the availability, approachability, and support and respect dimensions by using a total of 40-item five-point Likert scale (1 = strongly disagree, 2 = disagree, 3 = neutral, 4 = agree, and 5 = strongly agree) questions, and the pregnant women who scored ≥75% of the total were considered to have received good quality of prenatal care [13, 17]. Health facilities that had sphygmomanometers‚ fetoscopes‚ thermometers‚ speculums‚ measuring tapes‚ examination coaches‚ stethoscopes‚ blood pressure apparatuses, and adult weighing scales were considered to have made all the basic equipment as per the standard available [20]. Similarly, health facilities that have tetanus toxoid (TT) vaccine‚ iron folic with ferrous sulphate, and all other essential drugs as per the standard for a minimum stock of two months were also considered to have made essential drugs available.

### 2.4. Data Collection Tools and Procedures

A structured interviewer-administered questionnaire, chart reviews, and resource inventory checklists were prepared by reviewing articles [13, 17, 21–25]. The questionnaire was initially prepared in English and translated to Amharic and back to English for matters of consistency. Cronbach's alpha for all quality measurement items was 0.80. Exit interview was conducted among ANC follow-up women using the questionnaire. In addition, client charts were reviewed to identify received services, and resource checklists were completed to assess the availability of infrastructures in facilities.

### 2.5. Data Quality Control

A one-day training was given to four diploma and two BSc degree graduate nurse data collectors, and supervisors who had worked out of the study area were recruited. A pretest was conducted on 23 pregnant women at Tseda Health Center, and necessary modifications were made on the tool based on the findings. The data were checked daily by the principal investigator and the supervisors to maintain consistency and completeness.

### 2.6. Data Management and Analysis

Data were cleaned, coded, and entered into Epi-Info version 7 and exported to SPSS version 21 for analysis. Descriptive statistics, text narration, and tables were used to present the results. The binary logistic regression model was fitted for analysis. The multivariable logistic regression was used to adjust for confounders, and the variables with <0.2 *p* values during the bivariable logistic regression were entered into the multiple variable logistic regression analysis. Finally, an adjusted odds ratio (AOR) with a 95% confidence interval (CI) and less than 0.05 *p* values were used to declare factors associated with the outcome variable.

### 2.7. Ethics Approval

Ethical clearance was obtained from the Ethical Review Committee of the Institute of Public Health, College of Medicine and Health Sciences, University of Gondar. A permission letter was also obtained from the Amhara National Regional Health Bureau and the respective hospitals. Written informed consent was taken from each participant. Assent was taken from their parents for those participants aged below 18 years. Each eligible participant was informed about the purpose and the importance of the study. The participants were given an assurance that the confidentiality of information received at all levels will be maintained, and personal identifiers, like names, will not be used on copies.

## 3. Results

### 3.1. Sociodemographic and Economic Characteristics

A total of 465 pregnant women participated in the study with a response rate of 99.4%. The minimum and maximum ages of the participants were 16 and 47 years, respectively. The median age of the respondents was 25 years with the interquartile range (IQR) of 9 years. More than half (57.8%) of the participants were rural dwellers, and 14.6% attended higher institute education. More than three-fourths (76.0%) of the respondents were housewives (Table 1).

### 3.2. Obstetric History of the Pregnant Women

Nearly thirty percent (28.6%) of participants started their visits for their pregnancies of the moment in the third trimester. More than one-third (38.7%) and one-tenth (12.3%) of the pregnant women made their first and fourth visits, respectively. Furthermore, 17.0% of the prenatal care users had history of abortion, while 5.0% had history of still births (Table 2).

### 3.3. Organizational Infrastructures and Service Provision Factors

All of the health facilities had separate prenatal care counseling OPDs, but only 91.4% of the facilities had maternity waiting areas for services. Latrines, skilled prenatal care providers, clinical management guidelines, examination coaches, BP apparatuses, fetoscopes, weight scales, iron foliate, tetanus toxoid, treatment of syphilis, treatment of pregnancy-induced hypertension, deworming drugs, and HIV and HCG test kits were fully (100%) available during the data collection. Of the facilities, 86.5% had good access to electricity and clean water. Only half of the health facilities had hemoglobin test kits, while 86.0% had urine analysis, blood group, and stool test kits during data collection. Blood pressure, weight, fetal heart beat, and fetal positions of babies were checked for 99.4, 98.5, 79.8, and 70.5% of the pregnant women, respectively. In relation with laboratory investigations, 44.7, 69.2, and 92.9% had hemoglobin, blood group/RH, and HIV test services, correspondingly, and 96.1% received iron folate supplementation. Similarly, 35.5 and 68.0% of the women were informed about laboratory test results during prenatal care and place of delivery, respectively. In addition, 14.4, 94.6, and 15.9% of women had counselings about birth preparedness plans and proper nutrition and family planning methods (Table 3).

### 3.4. Quality of Prenatal Care

**Figure 1:**
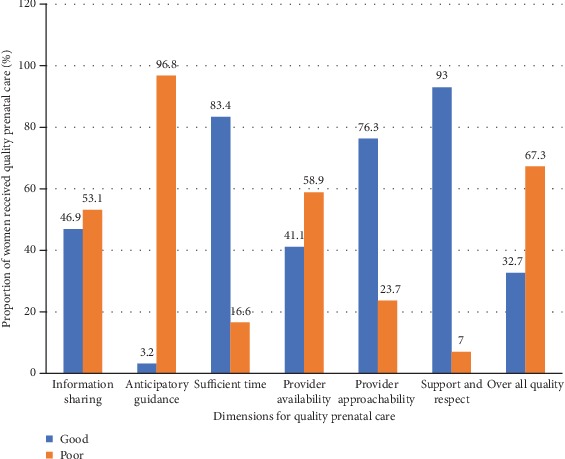
Magnitude of quality of prenatal care among pregnant women at Wogera district, Northwest Ethiopia, 2019 (*n* = 465).

This study revealed that the overall quality of prenatal care in the study area was 32.7% (95% CI: 28.1, 37.2). In addition, 46.9, 83.4, 93, and 3.2% of the prenatal care users shared adequate information, had sufficient time for discussion, received proper support as well as respect, and anticipatory guidance from the healthcare providers, respectively (Figure 1).

### 3.5. Factors Associated with Quality of Prenatal Care

Women who attained higher education were 2.9 times (AOR: 2.9; 95% CI: 1.03, 7.93) more likely to receive good quality prenatal care compared to women with no formal education. Pregnant women with >USD 175.5 monthly household income were 2.8 times (AOR: 2.8; 95% CI: 1.1, 7.8) more likely to receive high-quality prenatal care compared with those whose monthly household income was <USD 35.1. Women who attained ≥4 prenatal care services were 2.3 times (AOR: 2.3; 95% CI: 1.2, 4.4) more likely to receive good quality prenatal care than women who made one visit. Women who had prenatal care at health facilities with maternity waiting areas were 2.4 times (AOR: 2.4; 95% CI: 1.2, 5.0) more likely to receive good quality of care compared with women who went to facilities with no maternity waiting areas (Table 4).

## 4. Discussion

This study is aimed at assessing the quality of prenatal care provided to pregnant women at public health facilities and associated factors in Wogera district. Overall, 32.7% (95% CI: 28.1, 37.2) of the pregnant women received good quality prenatal care. The finding indicated that the majority of the pregnant women did not receive such care. This might predispose to women to home delivery and leads to poor birth outcomes.

The quality of prenatal care in this study was in line with that of a study done in Zambia (29%) [26] but was higher than the findings of studies conducted in Harar (24.3%) [13], Tigray (24.5%) [27], Bahir-Dar (27.6%) [9], Nepal (24%) [28], and Nigeria (4.6%) [29]. On the other hand, our finding was lower than those of studies conducted in Jimma (69.5%) [15]; Bahir Dar (47.7%) [23]; Lusaka, Zambia (47.1%) [30]; Kaki, Nepal (43%) [31]; and Sudan (38%) [32]. The variations might be due to differences in the quality of prenatal care measurement approaches, study designs, periods, settings, and cultures.

Nearly all of the pregnant women received blood pressure (BP) monitoring and weight assessments. This finding was high, but the fetal positioning (70.0%) and FHB (79.8%) assessment in this study were lower than the result of a study in Harari, Ethiopia [13]. Furthermore, the maternal advice given on nutrition and places of delivery which covered 94.6 and 68% of the targets, respectively, in our work was higher than similar advices on nutrition and birth preparedness plan that covered 35.3 and 14.4%, respectively, of the participants reported by a study in Gondar [33]. The quality of prenatal care and associated factors might differ due to variations in the availability of basic equipment, infrastructures, work load, and the commitment of prenatal care service providers resulting in inconsistencies in maternal healthcare information.

In this study, the majority (83.4%) of the respondents had sufficient time with healthcare providers. This finding was lower than those of studies done in Tigray (89.8%) and Lagos (94.6%) [27, 34]. The possible justification might be that the healthcare provider to client ratio was high in the other studies, resulting in a compromised quality of prenatal healthcare services.

Pregnant women with ≥USD 175.5 monthly household income were 2.8 times more likely to receive good quality prenatal care services compared with households earning <USD 35.1. This finding was in line with those of studies done in Nigeria and Nepal [28, 29]. The possible justification might be that high household income, perhaps, increases the ability of women to cover their travel expenses and easily access prenatal care services [35].

More educated women were 2.9 times more likely to get good quality prenatal care services than women with no formal education. This finding was consistent with those of studies done in Harar, Ethiopia [13]; Nepal [29]; and Nairobi [36]. The possible justification might be that education enables women to easily understand the importance of prenatal care services and empowers them to decide their healthcare service utilization. Similarly, educated women are better able to fully take all prenatal care services since they have good knowledge and favorable attitude towards prenatal care. Pregnant women who attended ≥4 prenatal care services were 2.3 times more likely to receive good quality care compared with women who take only one service. This finding was in line with those of studies done in Harari, Ethiopia [13], and Nigeria [29]. The possible justification might be that repeated exposure to prenatal care services might enhance the familiarity of the women with the services and encourages them to freely share information with healthcare providers.

Mothers who received prenatal care at health facilities with maternity waiting areas were 2.4 times more likely to receive good quality services compared with their counterparts. This finding was supported by a study done in the Gamo-Gofa zone [21]. The possible justification might be that getting prenatal care at health facilities that had maternity waiting areas might enhance their comfort and satisfaction at the time of service delivery during antenatal care follow-ups.

### 4.1. Limitation of the Study

This study lacked qualitative aspects in assessing the perceptions of clients and the opinion of experts. In addition, findings might also be subject to social desirability bias since interviews with mothers conducted were in the compounds of the health facilities.

## 5. Conclusion

The overall quality of prenatal care given for pregnant women was low. That is, the information sharing and anticipatory guidance dimensions were particularly poor. On the other hand, health care providers' support and respect and the provision of sufficient time for discussions with clients were relatively high. Variables such as household monthly income, maternal education, prenatal care visits, and the availability of maternal waiting areas were associated with the quality of healthcare delivered. Therefore, encouraging maternal education, building the capacity of healthcare providers, making basic infrastructures, and laboratory reagents available at healthcare facilities could improve the quality of care. Researchers had better use mixed methods, including qualitative aspects, to get a clear picture of the whole problem.

## Figures and Tables

**Table 1:** Sociodemographic and economic characteristics of pregnant women at Wogera district, 2019 (*n* = 465).

Variables	Category	Frequency	Percent (%)
Age (years)	<20	110	23.7
	20-25	126	27.1
	25-30	120	25.8
	>30	109	23.4

Residence	Urban	196	42.2
	Rural	269	57.8

Occupation	Housewife	355	76.0
	Civil servant	78	17.0
	Merchant	32	7.0

Maternal education	Unable to read and write	244	52.5
	Able to read and write	83	17.8
	Primary or secondary	70	15.1
	Higher education	68	14.6

Husband education	Unable to read and write	293	63.0
	Able to read and write	57	12.3
	Primary or secondary	46	9.9
	Higher education	69	14.8

Monthly household income (USD)	<35.1	60	12.9
	35.1-87.8	230	49.5
	87.8-175.5	117	25.2
	≥175.5	58	12.5

USD: United States Dollar.

**Table 2:** Obstetric history of pregnant women at Wogera district, 2019 (*n* = 465).

Variables	Category	Frequency (*n*)	Percent (%)
First initiation of prenatal care	First trimester	27	5.8
	Second trimester	305	65.6
	Third trimester	133	28.6

Number of prenatal care visit	First	180	38.7
	Second	139	29.9
	Third	89	19.1
	Fourth	57	12.3

History of abortion	Yes	80	17.0
	No	385	83.0

History of still birth	Yes	23	5.0
	No	442	95.0

Number of pregnancies	1	142	30.5
	2-5	271	58.3
	>5	52	11.2

**Table 3:** Service provision and organizational factors among pregnant women attending prenatal care at Wogera district, 2019 (*n* = 465).

Variables	History, physical examination, or laboratory performed	Frequency *n* (%)	Quality of prenatal care		*p* value
			Good	Poor	
Current obstetric history	Yes	464 (99.8)	152	312	—
	No	1 (0.2)	0	1	

Weight	Yes	458 (98.5)	151	307	0.32
	No	7 (1.5)	1	6	

Height	Yes	2 (0.4)	0	2	—
	No	463 (99.6)	152	311	

Blood pressure	Yes	462 (99.4)	152	310	—
	No	3 (0.6)	0	3	

Conjunctiva of the eye examined	Yes	454 (97.6)	151	303	0.23
	No	11 (2.4)	1	10	

Position of the baby assessed	Yes	328 (70.5)	107	221	0.96
	No	117 (29.5)	45	92	

Fetal heart beat checked	Yes	371 (79.8)	124	247	0.18
	No	94 (20.2)	28	66	

Edema checked	Yes	142 (30.5)	133	279	0.60
	No	53 (69.5)	19	34	

Vaginal examination	Yes	452 (97.2)	149	303	0.46
	No	13 (2.8)	3	10	

Hemoglobin/hematocrit	Yes	208 (44.7)	105	103	0.21
	No	257 (55.3)	47	210	

Rapid plasma reagin	Yes	315 (67.7)	144	171	0.38
	No	150 (32.3)	8	142	

Blood group	Yes	322 (69.2)	148	174	0.45
	No	143 (30.8)	4	139	

Rhesus factor	Yes	322 (69.2)	142	182	0.41
	No	143 (30.8)	10	131	

Urine analysis	Yes	314 (67.5)	134	180	0.33
	No	151 (32.5)	18	133	

Stool examination	Yes	97 (20.9)	61	36	0.27
	No	368 (79.1)	91	277	

Iron foliate supplementation	Yes	447 (96.1)	146	301	0.95
	No	18 (3.9)	6	12	

Mebendazole provision	Yes	133 (28.6)	131	2	0.43
	No	332 (71.4)	21	211	

Tetanus toxoid	Yes	427 (91.8)	121	306	0.52
	No	38 (8.2)	31	7	

**Table 4:** Factors associated with quality of prenatal care at public health facilities at Wogera district, 2019 (*n* = 465).

Variables	Category	Quality of prenatal care		Crude odds ratio (95% confidence interval)	Adjusted odds ratio (95% confidence interval)	*p* value
		Good	Poor			
Resident	Urban	72	124	1.4 (0.9, 2.0)	1.1 (0.7, 1.7)	0.801
	Rural	80	189	1	1	

Age (years)	<20	38	72	1	1	
	20-25	39	87	0.9 (0.5, 1.5)	0.6 (0.3, 1.1)	0.080
	25-30	43	77	1.1 (0.6, 1.8)	0.9 (0.5, 1.6)	0.670
	>30	32	77	0.8 (0.50, 1.4)	0.7 (0.4, 1.4)	0.387

Monthly household income (USD)	<35.1	12	48	1	1	
	35.1-87.8	63	167	1.5 (0.73, 0)	1.7 (0.8, 3.6)	0.180
	87.8-175.5	43	74	2.3 (1.1, 4.9)	1.9 (0.8, 4.4)	0.135
	>175.5	34	24	5.7 (2.5, 12.9)	2.8 (1.1, 7.8)	0.045

Abortion	Yes	36	44	1.9 (1.23, 1)	1.6 (0.96, 2.80)	0.070
	No	116	269	1	1	

Fetal heart beat checked	Yes	124	247	1.2 (0.7, 1.9)	1.5 (0.8, 2.6)	0.172
	No	28	66	1	1	

Maternity waiting area	Yes	295	130	2.8 (1.4, 5.4)	2.4 (1.2, 5.0)	0.017
	No	18	22	1	1	

Electricity	Yes	144	258	3.8 (1.8, 8.3)	2.2 (0.98, 5.1)	0.056
	No	8	55	1	1	

Number of prenatal visits	1	51	129	1	1	
	2	38	101	0.95 (0.6, 1.6)	0.8 (0.5, 1.5)	0.536
	3	34	55	1.6 (0.9, 2.7)	1.3 (0.7, 2.4)	0.396
	4+	29	28	2.6 (1.4, 4.8)	2.3 (1.2, 4.4)	0.014

Maternal education	No formal	83	244	1	1	
	Primary/secondary	27	43	1.9 (1.1, 3.2)	1.4 (0.8, 2.5)	0.301
	Higher	42	26	4.8 (2.7, 8.2)	2.9 (1.03, 7.93)	0.005

## Data Availability

All the data supporting the study findings are within the manuscript. Additional detailed information and raw data are available from the corresponding author on reasonable request.

## Abbreviations

ANC: Antenatal care
AOR: Adjusted odds ratio
CI: Confidence interval
ETB: Ethiopian birr
FHB: Fetal heart beat
SPSS: Statistical package for social science
WHO: World Health Organization
MMR: Maternal mortality ratio
MWA: Maternal waiting area.
